# Effect of Cochlear Implant Electrode Insertion Depth on Speech Perception Outcomes: A Systematic Review

**DOI:** 10.1097/ONO.0000000000000045

**Published:** 2023-12-14

**Authors:** Tabita M. Breitsprecher, Wolf-Dieter Baumgartner, Kevin Brown, Stefan Dazert, Una Doyle, Anandhan Dhanasingh, Wilma Großmann, Rudolf Hagen, Paul Van de Heyning, Robert Mlynski, Marcus Neudert, Gunesh Rajan, Kristen Rak, Vincent Van Rompaey, Joachim Schmutzhard, Stefan Volkenstein, Christiane Völter, Wilhelm Wimmer, Mario Zernotti, Nora M. Weiss

**Affiliations:** 1 Department of Otorhinolaryngology-Head and Neck Surgery, Ruhr-University Bochum, St. Elisabeth-Hospital Bochum, Bochum, Germany; 2 Universitätsklinik für Hals-, Nasen- und Ohrenkrankheiten, Medizinische Universität Wien, Wien, Austria; 3 Department of Otolaryngology/Head and Neck Surgery, University of North Carolina at Chapel Hill, Chapel Hill, NC; 4 MED-EL Elektromedizinische Geraete Gesellschaft m.b.H., Innsbruck, Austria; 5 Department of Translational Neurosciences, Faculty of Medicine and Health Sciences, University of Antwerp, Antwerp, Belgium; 6 Department of Otorhinolaryngology, Head and Neck Surgery, “Otto Körner,” Rostock University Medical Center, Rostock, Germany; 7 Department of Otorhinolaryngology, Plastic, Aesthetic and Reconstructive Head and Neck Surgery, Comprehensive Hearing Center, University of Würzburg, Würzburg, Germany; 8 Department of Otorhinolaryngology and Head & Neck Surgery, Antwerp University Hospital, Edegem, Belgium; 9 Department of Otorhinolaryngology Head and Neck Surgery, Technische Universität Dresden (oder TU Dresden), Faculty of Medicine (and University Hospital) Carl Gustav Carus, Dresden, Germany; 10 Otolaryngology, Head and Neck Surgery, Medical School, University of Western Australia, Perth, Australia; 11 Faculty of Health Sciences and Medicine, University of Lucerne, Luzern, Switzerland; 12 Department of Otorhinolaryngology-Head and Neck Surgery, Medical University Innsbruck, Innsbruck, Austria; 13 Department of Otorhinolaryngology, Head and Neck Surgery, Ruhr-University Bochum, Johannes Wesling Klinikum Minden, Bochum, Germany; 14 Department of Otolaryngology, Head and Neck Surgery, School of Medicine, Technical University of Munich (TUM), Munich, Germany; 15 Department of Otorhinolaryngology, TUM School of Medicine, Klinikum Rechts der Isar, Munich, Germany; 16 Division of Otolaryngology and Head and Neck Surgery, Sanatorio Allende, Catholic University of Córdoba and National University of Córdoba, Córdoba, Argentina; 17 International Graduate School of Neuroscience, Ruhr-University Bochum, Bochum, Germany.

**Keywords:** Angular insertion depth, Electrode insertion depth, Insertion angle, Speech perception

## Abstract

**Objective:**

The suitable electrode array choice is broadly discussed in cochlear implantation surgery. Whether to use a shorter electrode length under the aim of structure preservation versus choosing a longer array to achieve a greater cochlear coverage is a matter of debate. The aim of this review is to identify the impact of the insertion depth of a cochlear implant (CI) electrode array on CI users’ speech perception outcomes.

**Databases Reviewed:**

PubMed was searched for English-language articles that were published in a peer-reviewed journal from 1997 to 2022.

**Methods:**

A systematic electronic search of the literature was carried out using PubMed to find relevant literature on the impact of insertion depth on speech perception. The review was conducted according to the preferred reporting items for systematic reviews and meta-analyses guidelines of reporting. Studies in both, children and adults with pre- or postlingual hearing loss, implanted with a CI were included in this study. Articles written in languages other than English, literature reviews, meta-analyses, animal studies, histopathological studies, or studies pertaining exclusively to imaging modalities without reporting correlations between insertion depth and speech outcomes were excluded. The risk of bias was determined using the “Risk of Bias in Nonrandomized Studies of Interventions” tool. Articles were extracted by 2 authors independently using predefined search terms. The titles and abstracts were screened manually to identify studies that potentially meet the inclusion criteria. The extracted information included: the study population, type of hearing loss, outcomes reported, devices used, speech perception outcomes, insertion depth (linear insertion depth and/or the angular insertion depth), and correlation between insertion depth and the speech perception outcomes.

**Results:**

A total of 215 relevant studies were assessed for eligibility. Twenty-three studies met the inclusion criteria and were analyzed further. Seven studies found no significant correlation between insertion depth and speech perception outcomes. Fifteen found either a significant positive correlation or a positive effect between insertion depth and speech perception. Only 1 study found a significant negative correlation between insertion depth and speech perception outcomes.

**Conclusion:**

Although most studies reported a positive effect of insertion depth on speech perception outcomes, one-third of the identified studies reported no correlation. Thus, the insertion depth must be considered as a contributing factor to speech perception rather than as a major decisive criterion.

**Registration:**

This review has been registered in PROSPERO, the international prospective register of systematic reviews (CRD42021257547), available at https://www.crd.york.ac.uk/PROSPERO/.

The postoperative performance with a cochlear implant (CI) highly varies among patients. Patient-specific factors that are associated with postoperative speech perception are 1) the preoperative speech perception (1), 2) the duration of hearing loss (2,3), 3) the etiology of hearing loss (4), and 4) the age at implantation (5,6). Improvements in surgical techniques, electrode designs, and speech-processing strategies have led to outcome improvements (7,8). Implant-specific factors, such as the electrode-modiolar proximity have been discussed to influence postoperative speech perception. However, no clear beneficial association between postoperative speech perception and electrode position has been proven so far (9–12). A limitation of any systematic review is that most studies are heterogenous concerning confounding aspects such as the duration of deafness, residual hearing, sound coding strategy, educational level of the patients, wearing time of the audio processor, and level of rehabilitation. In studies reporting advantageous effects of deeper insertions, it is hypothesized that a deeper insertion of a CI electrode array into the apical region of the cochlea may enhance speech perception outcomes by improving the match between the programmed frequency bands of the electrode array and the tonotopic organization of the cochlea (13–16). Possible reasons for a poorer performance with deeper electrode array insertion (10) may be due to apical frequency pitch confusions caused by the close contact between the electrodes (17) or an increased insertion trauma (18). This systematic literature review aims to identify the association between insertion depth of CI electrode arrays and speech perception outcomes.

## METHODS

### Search Strategy

The review was conducted according to the preferred reporting items for systematic reviews and meta-analyses guidelines (19), using PubMed. Articles published from January 1997 to September 2022 were scanned. This period encompasses the first description of deep electrode array insertion in 1997 (20). Studies in children or adults with pre- or postlingual hearing loss implanted with a CI were included. Reviews, literature searches, meta-analyses, animal studies, cadaveric studies, studies not reporting speech performance outcomes, studies without correlation analysis, and studies in other languages than English were excluded. The review questions were based on the patient/population, intervention, comparison, outcome strategy (Table 1).

**TABLE 1.** PICO strategy

PICO	Criteria in review
Participants	Cochlear implant recipients (adults and children)
Intervention	Cochlear implantation
Comparator	None
Outcomes	Speech perception tests, insertion depth, correlation with insertion depth

### Study Selection

Search terms were: (“cochlear implant*”[Title] OR “electrode”[Title]) AND (“insertion depth”[Title/Abstract] OR “insertion angle”[Title/Abstract] OR “cochlear coverage” [Title/Abstract]). The abstracts were screened manually by 2 authors independently (T.M.B. and A.D.). Information on author(s), PubMed ID, publication year, study design, number of participants, etiology of hearing loss, age at implantation, type (prelingual or postlingual) and duration of deafness, type of CI electrode array, follow-up intervals, insertion depth, speech test outcome and correlation analyses were extracted from the articles.

### Risk of Bias Assessment

The risk of bias was estimated based on the Cochrane Collaboration Risk of Bias in Nonrandomized Studies of Interventions (ROBINS-I) tool for assessing risk of bias (21). The confounding factors were age at implantation (22,23), etiology of hearing loss (24,25), pre- versus postlingual deafness, listening experience (26,27), and the anatomy of the cochlea (“normal” vs “malformed”) (28).

## RESULTS

### Description of Studies

**FIG.1.**
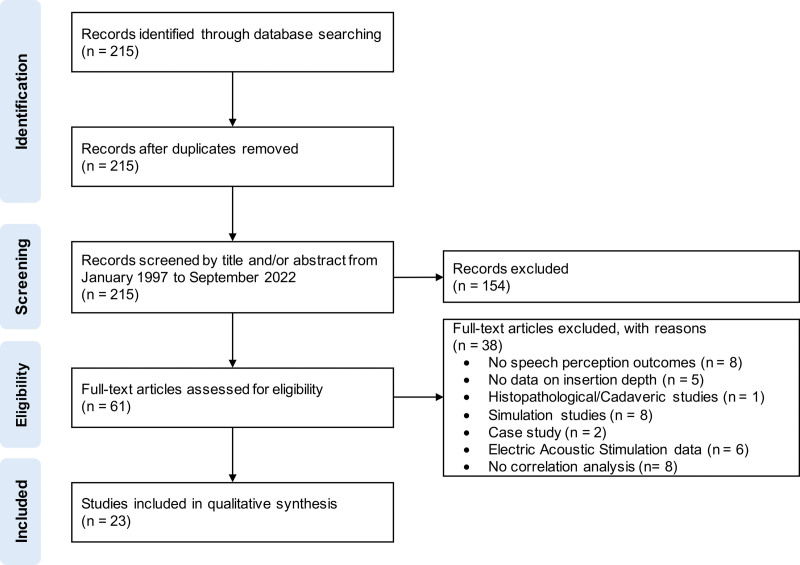
Flowchart showing the process of data acquisition and the number of identified studies according to the PRISMA (preferred reporting items for systematic reviews and meta-analyses) guidelines.

The database search revealed 215 studies that were assessed for eligibility (Fig. 1). A total of 154 studies were excluded after screening the abstract, and 61 full-text articles were assessed for eligibility. Further 38 articles that did not meet the predefined inclusion criteria were subsequently excluded. Twenty-three studies were included in the final systematic review. Studies were performed by 15 different CI centers.

### Demographics

Table 2 shows the participants’ demographic data from the 23 publications. One out of the 23 studies included children only, 1 study included children and adults and the remaining 21 studies included adults only. Eighteen studies were retrospective studies, whereas 5 included both, prospective and retrospective data.

**TABLE 2.** Demographic data

Author	Year	n	Electrode array type (LW/PM subgroups)	Mean age at implantation in years ± SD	Onset of hearing loss	Mean duration of deafness (years)	Residual hearing	Bilateral or unilateral	Follow-up period (months)	Statistical analysis	Association speech performance and insertion depth	Effect size
Canfarotta et al	2022	75 ears (adults)	Lateral wall: Med-El Flex28 (n = 28) Med-El FlexSoft/Med-El Standard (n = 47)	64.3 Med-El Flex28: 65.0 Med-El FlexSoft/ Med-El Standard 63.6	Postlingual	Med-El Flex28: 10.0; Med-El FlexSoft/Standard: 9.0	Yes	N/A	12	Pearson correlation	Positive	Medium
Fan et al	2022	29 ears (19 children, 10 adults)	Lateral wall: Shanghai Listent LCI20 PI (n = 29)	34.70 ± 7.33 (adults) 3.58 ± 1.77 (children)	N/A	Adults: 6.1 children: 1.1	N/A	Unilateral	6, 12	Multiple regression	6 months: positive 12 months: n.s.	Large –
Lo Russo et al	2022	50 ears (adults)	Lateral wall: Med-El Flex28 (n = 14) Med-El Flex24 (n = 1) Cochlear Nucleus CI422 (n = 2) Cochlear Nucleus CI522 (n = 1) Advanced Bionics HiFocus 1J (n = 4) Oticon Medical EVO (n = 4) Oticon Medical CLA (n = 7) Perimodiolar: Advanced Bionics HiFocus MS (n = 9); Cochlear Nucleus CI512 (n = 4); Cochlear Nucleus CI532 (n = 4)	60.5	Postlingual	Favorable outcome: 20.0 Unfavorable outcome: 22.0	No	Unilateral	6	Binary logistic regression model	n.s.	–
Heutink et al	2021	129 ears (adults)	Lateral wall: Cochlear Nucleus CI422/522 (n = 44) Perimodiolar: Cochlear Nucleus CI512/CI24RE (n = 85)	62.6 ± 13.0	Postlingual	25.0	N/A	Bilateral	14–92	Spearman correlation (Mann-Whitney *U*, Kruskal-Wallis test)	Positive	Small
Canfarotta et al	2021	50 ears (adults)	Lateral wall: Med-El Flex24 (n = 6) Med-El Flex28 (n = 6) Med-El Standard (n = 29) Perimodiolar: Cochlear Contour Advance (n = 7) Advanced Bionics HiFocus MS (n = 2)	60.4 ± 14.7	Postlingual	N/A	Yes	Bilateral	6	Simple linear regression	n.s.	–
Canfarotta et al	2021	19 ears (adults)	Lateral wall: Med-El Medium (n = 6) Med-El Standard (n = 13)	Med-El Medium: 63.0 Med-El Standard: 61.0	Postlingual	Med-El Medium: 5.0 Med-El Standard: 4.0	Yes	N/A	1, 3, 6, 12, 24,48	Linear mixed models	Positive	Small
Nassiri et al	2020	24 ears (adults)	Perimodiolar: Cochlear Nucleus CI532 (n = 24)	67.7	Postlingual	2.7	Yes	N/A	6, 12	Multivariate regression	Positive	N/A
Kuthubutheen et al	2019	55 ears (adults)	Lateral wall: Med-El FlexSoft (n = 34) Med-El Flex28 (n=21)	62.0	Postlingual	N/A	No	N/A	6	Mann-Whitney *U* test and Chi-square-analyses	n.s.	–
Chakravorti et al	2019	220 ears (adults)	Lateral wall: Advanced Bionics HiFocus 1J (n = 29) Cochlear Nucleus CI422 (n = 20) Cochlear Nucleus CI522 (n = 11) Med-El Flex24 (n = 3) Med-El Flex28 (n = 22) Med-El Medium (n = 1) Med-El Standard (n = 24) Perimodiolar: Cochlear Contour Advance (n = 89) Advanced Bionics HiFocus MS (n = 21);	Lateral wall: 56.0 Perimodiolar: 57.1	Postlingual	N/A	N/A	N/A	2, 192	Stepwise multiple regression (Pearson)	LW: positive PM: n.s.	LW: N/A PM: –
Doubi et al	2019	162 ears (children)	N/A Electrodes from Advanced Bionics, Cochlear, Med-El	3.0	Prelingual	N/A	No	Unilateral (n = 32) bilateral (n = 65)	6, 12, 36	group comparisons by *t* test	n.s.	–
Helbig et al	2018	91 ears (adults)	Lateral wall: Med-El Flex20 (n = 7) Med-El Flex24 (n = 28) Med-El Flex28 (n = 32) Med-El FlexSoft (n = 24)	58.3	Postlingual	N/A	Yes	N/A	12	Pearson correlation	Positive	Small
O’Connell et al	2017	48 ears (adults)	Lateral wall: Med-El Flex24 (n = 4) Med-El Flex28 (n = 28) Med-El Standard (n = 14)	N/A	Postlingual	N/A	Yes	N/A	6, 18	Pearson or Spearman correlation	Positive	Medium
Büchner et al	2014	97 ears (adults)	Lateral wall: Med-El Flex20 (n = 23) Med-El Flex24 (n = 25) Med-El Flex28 (n = 35)	62.81	Postlingual	0.92	Yes	unilateral (n = 85) bilateral (n = 6)	3, 6	Group comparison by Mann-Whitney *U* test	Positive	N/A
Hilly et al	2016	120 ears (adults)	Lateral wall: Advanced Bionics HiFocus 1J (n = 120)	52.6	N/A	N/A	N/A	N/A	12	Nonparametric tests and Spearman correlation	Positive	Medium
O’Connell et al	2016	20 ears (adults)	Lateral wall: Cochlear CI422 (n = 20)	63.2 ± 15.5	Postlingual	N/A	Yes	N/A	6, 18	Pearson correlation	Positive	Small
O’Connell et al	2016	220 ears (adults)	Lateral wall: Med-El Flex28 (n = 28) Med-El Standard (n = 18) Med-El Flex24 (n = 4) Med-El Medium (n = 1) Advanced Bionics HiFocus 1J (n = 21) Cochlear Nucleus CI422/522 (n = 19) Perimodiolar: Cochlear Contour Advance (n = 115) Advanced Bionics HiFocus MS (n = 14)	60.2	Postlingual	N/A	N/A	N/A	12, 16	Pearson or Spearman correlation, multivariate regression	Positive	CI422: large Others: n.s.
van der Jagt et al	2016	206 ears (adults)	Lateral wall: Advanced Bionics HiFocus 1J (n = 110) Perimodiolar: Advanced Bionics HiFocus MS (n = 96)	Lateral wall: 38.8 Perimodiolar: 47.4	Postlingual	LW: 28.0 PM: 16.0	Yes	Unilateral	1, 3, 6	Linear mixed model analysis	n.s.	–
Van der Marel et al	2015	203 ears (adults)	Lateral wall: Advanced Bionics HiFocus 1 Advanced Bionics HiFocus 1J	Prelingual: 39 Postlingual: 56	Pre- and postlingual	Prelingual: 37 ± 12 Postlingual: 22 ± 18	N/A	Unilateral	12, 24	1- and 2-tailed t-tests with Bonferroni correction	n.s.	–
Esquia Medina et al	2013	25 ears (22 adults)	Lateral wall: Med-El Standard Med-El Flex EAS Med-El FlexSoft	55.0	Postlingual	14.2	N/A	Unilateral (n = 22) bilateral (n = 3)	6, 12	Simple linear regression	Positive	Large
Finley et al	2008	14 ears (adults)	Lateral wall: Advanced Bionics HiFocus 1 Advanced Bionics HiFocus 1J Perimodiolar: Advanced Bionics HiFocus Helix	60.3	N/A	11.7	N/A	N/A	2, 4, 36	Bivariant and partial correlations (single-tailed)	Negative	N/A
Yukawa et al	2004	48 ears (adults)	Lateral wall: Cochlear Nucleus 22	52.0	Postlingual	7.3	N/A	N/A	6	Multiple regression with CUNY in noise as dependent variable.	Positive	N/A
Skinner et al	2002	13 ears (adults)	Lateral wall: Cochlear Nucleus 22	50.5	Postlingual	9.1	N/A	Unilateral	3, 6, 8, 12	Simple linear regression	Positive	Medium
Hodges et al	1999	31 ears (adults)	Lateral wall: Cochlear Nucleus 22	63.1	Postlingual	5.6	N/A	N/A	6	Spearman correlation	n.s.	–

The etiology of hearing loss over all studies was heterogenous. The majority of studies did not specify whether the participants had uni- or bilateral hearing loss (13 studies). Five studies included only patients with unilateral hearing loss, 2 studies included exclusively patients with bilateral hearing loss and 3 studies reported inclusion of both, uni- and bilateral hearing loss. The age at implantation was reported in all studies. The duration of deafness was reported in 14 studies whereas the remaining 9 studies did not indicate the duration of deafness prior to implantation. The majority (13 studies) included postlingually deafened subjects only, whereas 1 study reported on patients with prelingual deafness and 3 studies included both pre- and postlingual deaf CI users. In 4 studies the onset of hearing loss was not reported. Regarding residual hearing prior surgery, 9 studies reported residual hearing in CI candidates, whereas in 3 studies no functional residual hearing was reported and in 11 studies information about the residual hearing was not provided.

The devices included originated from 5 different manufacturers: Advanced Bionics (Valencia, CA), Cochlear Corporation (Sydney, NSW, Australia), Oticon (Somerset, NJ), Med-El Medical Electronics (Innsbruck, Austria), and Shanghai Listent (Shanghai, China). Table 3 shows the different electrode array types and lengths used in the reviewed studies.

**TABLE 3.** Electrode array types and lengths

Electrode type	Device name	Length (mm)	Manufacturer
Perimodiolar electrodes	Slim Modiolar (CI532)	18.4	Cochlear Corporation
	Contour Advance	19.2	Cochlear Corporation
	HiFocus MidScala	23.7	Advanced Bionics
	HiFocus Helix	24.5	Advanced Bionics
Straight electrodes	LCI20	20.0	Shanghai Listent
	Flex24	24.0	MED-EL Medical Electronics
	Slim Straight	20.0–25.0	Cochlear Corporation
	Medium	24.0	MED-EL Medical Electronics
	HiFocus 1	24.5	Advanced Bionics
	HiFocus 1J	25.0	Advanced Bionics
	EVO	25.0	Oticon
	CLA	26.0	Oticon
	Flex28	28.0	MED-EL Medical Electronics
	FlexSoft	31.5	MED-EL Medical Electronics
	Standard	31.5	MED-EL Medical Electronics

### Speech Perception

Depending on the origin of the study and the age of participants different speech materials in quiet and in noise for monosyllables (eg, consonant-vowel-consonant [CVC]); Freiburger monosyllable test, multisyllables, and sentences (eg, clinical assessment of pragmatics; speech intelligibility rating; hearing in noise test [HINT]; Oldenburger sentence test; City University of New York; Kowal-Bench speech-in-noise) were used.

### Insertion Depth

Fourteen studies measured the angular insertion depth (AID) in degrees (11,29–41). In one of the studies, the AID was presented graphically (10). One of the studies (42), reported the outcomes as percentage coverage (Group A ≥85% vs Group B ≤85%). Two studies presented the linear insertion depth (LID) in mm (43,44). Five studies reported both AID in degrees and LID in mm (9,13,22,45,46).

**FIG. 2.**
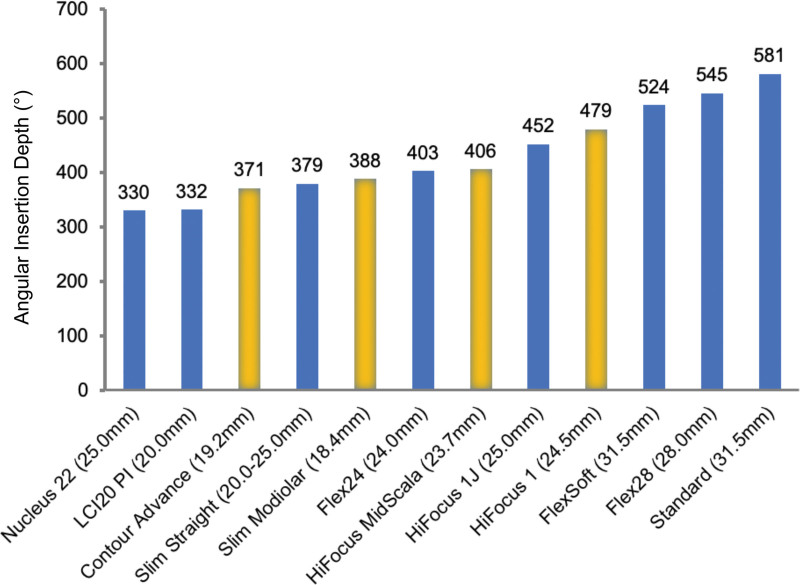
Angular insertion depth of the individual electrode array types and lengths. Blue bars: lateral wall electrode arrays; yellow bars: perimodiolar electrode arrays; number above bars: mean AID (°) of individual electrode array types among reported studies.

The mean AID reported across all studies in this review ranged between 330° and 608° and the mean LID ranged between 18.9 mm and 24.3 mm. Figure 2 shows the AID of the individual electrode array types and lengths reported amongst the different studies.

### Correlation Between Speech Perception and Insertion Depth

Seven studies found no significant correlation between insertion depth and speech perception outcomes (9,22,31,37,41,42,44).

Fifteen studies reported a positive effect or significant positive correlation between insertion depth and speech perception (11,13,29,30,32–36,38–40,43,45,46) (Table 2). Among these studies, 3 studies showed heterogenous results depending on the inserted electrode array type, the selected speech recognition test, or the follow-up period (34,36,38). The effect size of these studies was evaluated by 1) linear correlation (Pearson’s r, Spearman’s rank), 2) linear mixed models, 3) group comparison (*t* test, Mann-Whitney *U* test), or 4) by regression analysis (uni- and multivariant). Three studies found a strong positive correlation between insertion depth and speech perception (32,36,45), yet O’Connell et al (32) showed this effect only for 1 out of 2 tested electrode array types Fan et al (36) noticed the strong correlation only for multisyllabic word or sentence tests, but not in monosyllabic word recognition. One study found a significant negative correlation between insertion depth and speech perception outcomes (10).

Seven studies compared the effect of insertion depth on speech perception using different types of electrode arrays, that is lateral wall versus perimodiolar. Although Doubi et al (42), used electrode arrays from various manufacturers, they did not list the types nor analyze the speech perception outcomes for the specific array types. Lo Russo et al (22) reported that apart from the surgical insertion depth no other position-related variables were significantly different between the patients that had a “favorable” outcome versus an “unfavorable” outcome and that the perimodiolar versus lateral wall design had no influence on speech perception. Heutink et al (35), showed that speech perception was higher in the perimodiolar electrode array group that had relatively deeper insertion depth than in the lateral wall electrode array group. Likewise, Canfarotta et al (31) showed that both, array design and interaction of array design and AID significantly contribute to speech perception. The results of Chakravorti et al (38) also confirmed the electrode array position as a significant factor in audiological outcomes. The most significant positional factors that predicted the outcome for perimodiolar arrays were a full ST insertion and the modiolar distance, whilst for the lateral wall arrays it was the depth of insertion. The O’Connell et al (34) study, indicated that significant differences in AID were observed as a function of electrode array type, with lateral wall electrode arrays having a deeper insertion compared to perimodiolar electrode arrays. The AID was significantly correlated with the CNC scores. The electrode array type had no significant correlation with the CNC scores or the AzBio scores in the study. The scalar electrode array location had a significant effect on the CNC and the AzBio scores.

### Quality of Studies

**FIG. 3.**
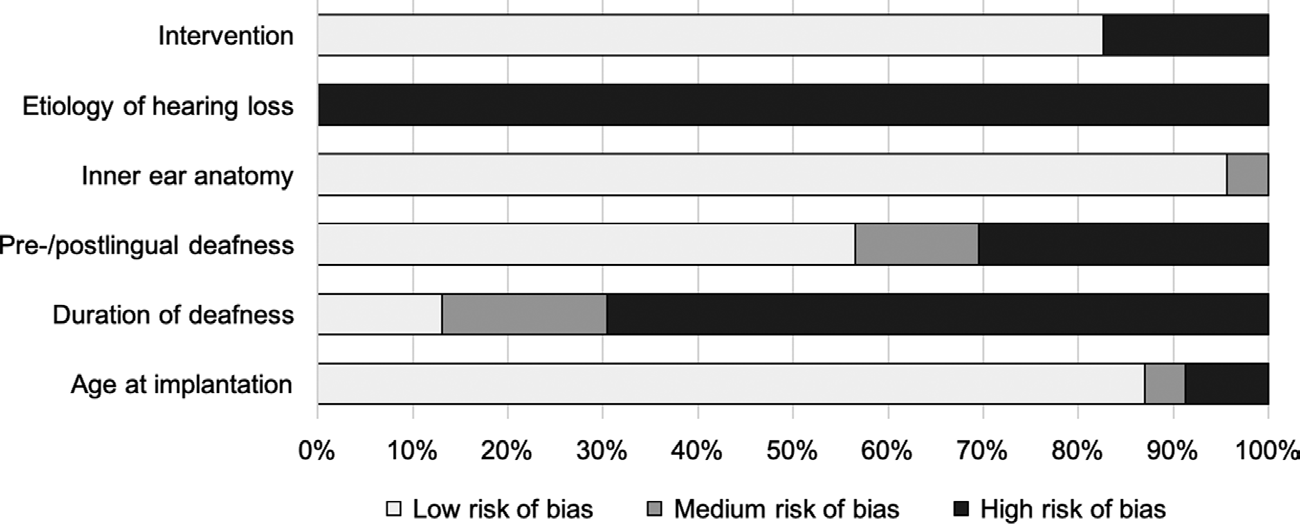
Risk of bias assessed using the Risk of Bias in Nonrandomized Studies of Interventions (ROBINS-I) tool.

The overall risk of bias of the studies as determined using the ROBINS-I tool was medium to high. The cumulative risk of bias is shown in Figure 3.

### Meta-Analyses

Heterogeneity of audiological outcomes precluded meta-analyses of the correlation data.

## DISCUSSION

### Electrode Array Design

Confounders that may influence the insertion trauma and thus the postoperative speech perception are the type of the electrode array (perimodiolar versus lateral wall) and the insertion site (scala tympani [ST] vs scala vestibuli [SV]) as well as the proximity to the modiolus (33,34,47,48). O’Connell et al (34) and Chakravorti et al (38), divided their data analyses depending on the different array types (lateral wall versus perimodiolar) and found a significant difference in the AID between these electrode array types in favor of lateral wall electrode arrays. However, the CNC scores were not significantly associated with the electrode array type although the overall AID was correlated significantly with the CNC scores. In this review, the mean AID across all studies was greater for the lateral wall electrode arrays compared to perimodiolar electrode arrays. Interestingly, there was no clear association between the electrode length and the AID. This effect may be explained by variances in the CDL assessment among the studies (49). Another explanation may be that shorter electrode arrays were chosen for smaller CDL, thus leading to relatively deeper AID (45). One explanation why a deep insertion may confer a benefit in speech perception is that greater insertion depth may improve the match between the tonotopic frequency and electric stimulation from the CI electrode arrays (11,13,29,30,34–36,39,40,43,45,46). A greater AID is assumed to cover a greater number of the spiral ganglion cells in the cochlea since they may extend up to 630° to 720° (16,50). Interestingly, insertion depths exceeding 720° are not associated with better hearing outcomes so far (51). This might be caused by technical limitations, since the lowest detectable frequency through the default settings of all tested audio processors is 70 Hz (52). However, it has to be considered that frequencies beyond 70 Hz likely do not influence speech perception outcomes since the speech frequency band ranges from 0.5 kHz to 4 kHz (53). Consequently, a benefit from perceiving these frequencies has to be expected rather for the localization of sound than for speech perception. According to mathematic models based on calculation models from Stakhovskaya et al (54), in an angular-based spiral ganglion map, a 720° insertion covers a corresponding frequency of 21 Hz, which is lower than the processor’s detection range (55). This review reports positive correlations between insertion depth and speech perception more frequently in studies using lateral wall electrode arrays independently from the CI manufacturer and in 1 study using perimodiolar electrode arrays (11), while those reporting no effect (9,22,31,37,38,41,42), or a negative effect used both, lateral wall and perimodiolar arrays (10). By Esquia Medina et al (45) the postoperative AID was considered in relation to the cochlear perimeter (CP) (AID/CP ratio). The speech perception was then correlated to the AID/CP ratio. Nonetheless, there was a significant positive correlation between the AID/CP ratio and the monosyllabic word scores at 6 months since deep insertions in small cochleae appeared to yield smaller electrode-modiolus distances and better hearing performance. However, at 12 months there was no significant correlation. Hilly et al (40) showed that 1 year after cochlear implantation an insertion depth of less than 1 turn had significantly lower HINT scores compared to an insertion depth of more than 1 turn. However, the depth of insertion as a continuous variable versus the HINT scores only showed an observational trend towards a positive effect leading to the assumption, that a certain cutoff value for the insertion depth is decisive for postoperative speech perception. Finley et al (10) found that the basal AID had a significant negative correlation with the (CNC) score, but not when age was considered a confounding factor. A shift from a middle to basal insertion pattern was also significantly negatively correlated. They assume a reduced stimulation of the basal turn, particularly the hook region due to the potential overinsertion since the basal electrode may be pushed towards the lateral wall by a deeper insertion of the electrode array (10). Hochmair et al (56), compared stimulation of the basal 8-electrode contacts of a 12-electrode contact array to a full stimulation of all 12-electrode contacts and found a better speech perception (monosyllabic words and sentence test) when stimulating all electrode contacts. However, distinguishing between the effect of insertion depth and the effect of the number of electrodes stimulated may not be assessed in this study design. Buchman et al (57) compared the postoperative speech perception of CI recipients implanted with a medium-length electrode array (24.0 mm) and standard-length electrode array (31.5 mm) in a prospective randomized trial. They reported a better speech performance at 12 months in subjects implanted with standard-length electrode arrays (mean AID: 657°) compared to the medium-length electrode arrays (mean AID: 423°).

### Quality of Hearing

An association between deeper insertion and frequency discrimination is frequently discussed (58–60). According to Peters et al, the pitch perception with a CI is 1 to 2 octaves lower than the predicted pitch. No significant correlation between the mismatch and the CVC phoneme recognition score was found comparing a perimodiolar electrode array (CI512, Contour Advance) to a lateral wall electrode array (CI422, Slim Straight) (59,61–63). In contrast, Canfarotta et al (15), report better CNC scores with smaller mismatch and greater angular separation between the contacts during the first 6 months of device use. In most studies investigating the quality of hearing at 6 and 12 months post-CI surgery, longer experience with the CI seemed to diminish the negative effect of a high frequency to place mismatch (15,36,60).

### Surgical Approach

O’Connell et al (34) report that the round window/extended round window approach was more likely to result in ST insertion compared to cochleostomy, and that ST insertions are associated with significantly better CNC outcomes compared to SV insertions. Several other sources indicate a better speech perception with ST insertions compared to SV insertion (7,10,34,46,64,65). In contrast, Ketterer et al (66), did not find a significantly higher rate of dislocation in cochleostomy-inserted arrays. However, it is hypothesized that speech perception is influenced by dislocations into SV (13). Skinner et al (46) report 1 patient with an electrode insertion near the oval window into the SV. This patient showed no improvement in word scores over a number of years indicating deleterious effects of SV insertion. The negative effect between insertion depth and speech perception reported by Finley et al (10) may be explained by a high rate of deeper insertions in cases of SV insertion in this study. The study used a cochleostomy approach and had a high rate of SV insertions and scalar translocations. All of these aspects have been associated with cochlear trauma and poorer speech perception outcomes in the past, so the negative effect may rather be caused by these surgical aspects than by AID (65,67–69). Thus, the AID in this study is biased by the traumatic approach.

### Limitations

Since some studies were performed by the same CI center in a comparatively short period of time, overlaps in cohorts—and in consequence findings—cannot be ruled out with complete reliability. Nevertheless, studies conducted in the same departments mostly investigated different electrode types, indicating that different cohorts were included.

As a further limitation, the effect of time and the duration of rehabilitation were not assessed in this review. The follow-up period may influence speech perception since the CI users gain additional listening experience over time and may profit from longer rehabilitation periods. Standard speech scores can improve for up to 3–5 years postimplantation in children and adults (70,71). A systematic review by Heutink et al (12) reports performance plateaus within 1 year after implantation. Altogether, any studies are heterogenous concerning confounding aspects such as the duration of deafness, the etiology of hearing loss, residual hearing, sound coding strategy, the educational level of the patients, the daily wearing time of the audio processor, and the level of rehabilitation. Consequently, these aspects could not systematically be assessed in this review. Further, the cochlear size may affect both, the AID and the electrode-modiolus proximity (11). Consequently, a conclusion whether a positive effect of deeper insertion is rather caused by the AID or a closer contact of the electrode to the modiolus cannot finally be drawn.

## CONCLUSION

Although, a majority of studies in this review showed a positive effect of the insertion depth on speech perception, confounders and contradictory evidence exist that hinder determining specific factors that affect the postoperative speech perception outcome. Patient-specific parameters are likely to play a strong role. To fully address the issue adequately in the future, the effect of insertion depth on speech perception needs to be investigated in controlled prospective studies.

## FUNDING SOURCES

None declared.

## CONFLICT OF INTEREST STATEMENT

None declared.

## DATA AVAILABILITY STATEMENT

The datasets generated during and/or analyzed during the current study are not publicly available, but are available from the corresponding author on reasonable request.
